# Coercive Parenting and Adolescent Developmental Outcomes: The Moderating Effects of Empathic Concern and Perception of Social Rejection

**DOI:** 10.3390/ijerph17103538

**Published:** 2020-05-19

**Authors:** Spencer De Li, Yiwei Xia, Ruoshan Xiong, Jienan Li, Yiyi Chen

**Affiliations:** 1 Department of Sociology, University of Macau, Macao 999078, China; spencerli@um.edu.mo (S.D.L.); ljn_0627@163.com (J.L.); chenyiyi1062@163.com (Y.C.); 2 School of Law, Southwestern University of Finance and Economics, Chengdu 611130, China; xiayw@swufe.edu.cn

**Keywords:** coercive parenting, developmental outcomes, empathic concern, perception of social rejection

## Abstract

Previous studies have identified coercive parenting as a prevalent parenting style in Chinese society. They suggested that this style of parenting could promote prosocial behavior and school commitment when combined with close monitoring and proper training, but it could also undermine mental health. This study critically examines these claims. Based on the existing theory and research, it is predicted that the influences of coercive parenting on adolescent development vary according to adolescent personal attributes including empathic concern and perception of social rejection. Through the analysis of two-wave survey data collected from a probability sample of 1085 Chinese adolescents, this study found that adolescents with higher levels of empathic concern and perceived social rejection reported less delinquency and stronger school commitment than their peers with lower levels of such attributes, when coercive parenting was low to moderate. However, under the condition of excessive coercive control, these adolescents demonstrated more delinquency and weaker school commitment. Empathic concern and perception of social rejection, on the other hand, played no or limited role in moderating the relationship between coercive parenting and depression. These results suggest that the influences of coercive parenting are dynamic and are subject to change as they interact with adolescent personal characteristics across different developmental domains.

## 1. Introduction

Coercive parenting is an emerging concept that has drawn increasing attention in empirical research. Prior research suggested that Chinese parents tend to use coercive strategies more often than their Western counterparts when setting and enforcing rules with their children [1,2]. In the Chinese context, this parenting style has shown conflicting effects. On the one hand, it may promote school commitment, academic achievement and social conformity, when combined with close monitoring and proper training [3]. On the other hand, coercive parenting may undermine children’s psychological wellbeing because of its over-reliance on psychological and physical control [4,5].

Despite the high prevalence of coercive parenting in Chinese society as suggested in prior research [6], few studies have examined how this parenting style influences developmental outcomes in society. Prior research on coercive parenting often takes a comparative approach to contrast this style of parenting with the other styles that are more prevalent in the West (e.g., authoritative parenting) and emphasizes its general characteristics and functions in Chinese society. What is lacking is the analysis of its contextualized effects within Chinese communities. This study attempts to fill these gaps in empirical research. Using two-wave survey data collected from a probability sample of 1085 secondary school students in China, we try to determine how coercive parenting affects adolescents who possess different levels of empathic concern and perception of social rejection, two affective and cognitive attributes frequently observed among Chinese adolescents [7,8]. To provide a broader assessment of the consequences of coercive parenting, we examine its influences across three key developmental outcomes, including delinquency, school commitment, and depression.

Based on the existing literature, we believe that coercive parenting in general would undermine healthy development. However, adolescents with high level of empathic concern may have more ability to sympathize with their parents and learn to cope with parental inconsistency and harsh treatment as long as parental coercive behavior remains at a tolerable level. Similarly, youths who have experienced greater social rejection could be more accepting of coercive parenting because, while living with coercive parents might be stressful, it is more beneficial than being socially isolated. Moreover, studies have shown that Chinese children sometimes interpreted parents’ attempts to control them through coercive behavior as caring acts [9,10]. For these reasons, the impact of coercive parenting may not be consistently positive or negative with regard to any of the developmental outcomes. Rather, its effect may vary depending on personal characteristics including adolescent empathic concern and perception of social rejection.

### 1.1. Coercive Parenting and Its Practices in Chinese Communities

In psychological research, the main characteristics of coercive parenting are defined as excessive use of coercive tactics such as domination, intimidation, and humiliation to promote obedience and success, with less concern for the child’s emotional wellbeing [2,11,12,13]. Although disagreements exist about the prevalence of coercive parenting in Chinese communities, most studies indicate that Chinese parents tend to be more restrictive and control-oriented and are less likely to show overt warmth than European or European-American parents [14]. Researchers attribute Chinese parents’ coercive parenting practice to the Confucian tradition that emphasizes the pursuit of group goals, interdependence, and children’s responsibility to be obedient to their parents [15]. In spite of the evidence of cultural changes as a result of unprecedented globalization that have led to increased adoption of Western childrearing philosophy and practices in China in the last few decades [16], much of the East-West divide formed by cultural adaptation to the local environment in an evolutionary process over thousands of years still remains [17]. Consequently, Chinese parenting practices continue to be more coercive and restrictive when compared with the prevailing norms of parenting in the West.

### 1.2. Coercive Parenting and Adolescent Developmental Outcomes

Coercive parenting has generally been identified as a key predictor of a range of negative developmental outcomes among children and adolescents, including delinquent behavior, mental health problems and poor academic performance [4,18,19]. A sizeable body of research has suggested that coercive parenting predicts adolescent maladjustment in both psychosocial and academic domains [20,21]. For example, drawing on two-wave longitudinal data collected from 601 adolescents in three Australian schools, Rowe et al. [22] found that coercive parenting was positively related to social anxiety, feeing of loneliness, depressive symptoms, and behavioral difficulties. Prior research has suggested that hostility and psychological control are two important components of coercive parenting [23]. In the context of parenting behavior, hostility refers to overt verbal and physical aggression such as physical punishment, hitting, shouting and scolding by parents in an attempt to pressure children to fulfil their high or unrealistic expectations. Psychological control, in contrast, is covert aggression and intrusive control that parents impose on children in order to manipulate their behaviors and cognitions by coercive disciplinary strategies such as threats, induction of guilt and shame, love withdrawal, and authority assertion [24]. While parental hostility and psychological control have been associated with a similar set of developmental problems, the latter has a much more detrimental effect on adolescent wellbeing [25]. Research has shown that coercive parents’ use of psychological control interferes with adolescents’ development of autonomy and independence, resulting in adolescent psychosocial maladjustment and academic difficulties [26,27].

Consistent with studies conducted in the West, several studies have shown that coercive parenting is associated with negative developmental outcomes in Chinese society [4,5]. Unlike Western parents who typically emphasize the development of autonomy and psychosocial wellbeing, Chinese parents place more value on children’s academic and social achievement [28]. They often use power-coercive tactics, especially psychological control and physical discipline, to compel their children to follow strict rules and to strive for academic success [6]. In general, these coercive parenting practices have been related to a host of internalized and externalized difficulties in Chinese adolescents, such as depression, low self-esteem, lack of self-confidence, academic difficulties, and delinquent behavior [29,30]. However, it should be noted that the influences of coercive parenting have not been shown to be consistently negative in Chinese society. Some studies indicated that coercive control had no or less negative effect among Chinese children compared to children in Western societies, especially when it was combined with a reasonable amount of guidance and supervision [9,10].

### 1.3. The Moderating Roles of Empathic Concern

Coercive parenting does not influence all children the same way. Children raised by coercive parents are not all poorly adjusted, as children with different individual characteristics tend to be differentially vulnerable to the influence of coercive parenting. The social ecological theory [31] provides a general framework to explain individual differences in the development of psychosocial problems under coercive parenting. According to this theory, adolescent developmental problems arise from complex interactions between individuals and the multiple systems in which they are embedded, such as family, school and neighborhood [32]. As an external stressor, coercive parenting likely interacts with other individual and social factors to influence adolescent developmental outcomes. One of these factors could be empathic concern. Defined as feelings of compassion and concern for others in need, empathic concern is an affective aspect of empathy [33]. As empathic concern might have been well developed before puberty, it can function as a stable disposition across adolescence [34,35]. In general, empathic concern has been linked to positive developmental outcomes, including prosocial behavior [36], decreased levels of conduct disorder [37] and academic achievement [38]. For example, previous studies have shown that when adolescents are more compassionate and more understanding of others’ feelings and actions, they are more likely to be committed to prosocial behaviors aimed at helping others, thereby reducing the likelihood of engaging in delinquent behavior [39].

The influence of empathic concern, however, may not be consistently positive among children and adolescents exposed to varying levels of coercive parenting. The differential susceptibility hypothesis provides a useful way to understand the different patterns of interplay between coercive parenting and empathic concern in relation to adolescent adjustment [40]. According to this perspective, individuals vary in their developmental plasticity and susceptibility to environmental influences. People who are more “plastic” or malleable are more responsive to both supportive and unsupportive environmental conditions [41]. In this regard, adolescents with temperamental or emotional susceptibilities (e.g., empathic concern) not only manifest more negative outcomes when exposed to adverse circumstances (e.g., high levels of coercive parenting), but are also more likely to develop better outcomes when exposed to positive environments including the absence of adversity (e.g., low levels of coercive parenting) compared to those without the susceptibilities. Therefore, instead of operating exclusively as a protective or risk factor, empathic concern might function as a plasticity factor, yielding positive developmental outcomes under the condition of low coercive parenting, but negative outcomes when co-occurring with high levels of coercive parenting. Consistent with the propositions of the differential susceptibility hypothesis, a growing number of studies have shown that children higher in sensitivity to social contexts are more susceptible to both negative and supportive rearing environments [42,43]. For example, in support of the differential susceptibility hypothesis, Davis, Luce and Davalos [44] found that, compared to their counterparts lower in empathic concern, adolescents who possessed higher empathic concern were more heavily involved in prosocial behavior when exposed to fewer negative life events, but were less likely to exhibit prosocial behavior when exposed to more negative life events. Similarly, in their empirical test of the interpersonal acceptance-rejection theory, Carrasco et al. [45] found that children’s emotional dependence mitigates the impact of parental rejection on adolescent psychological maladjustment, including impaired self-esteem, emotional instability, and hostility and aggression when they experienced a moderate level of the stressor. However, when parental rejection reaches a high level, adolescents would develop more externalized and internalized problems.

### 1.4. The Moderating Roles of the Perception of Social Rejection

Another related factor that could potentially condition the relationship between coercive parenting and adolescent developmental outcomes is the perception of social rejection, defined as an individual’s cognitive appraisal of the extent to which he or she is rejected by others in social relationships [46]. As a relatively stable personality trait across adolescence, perception of social rejection has shown a positive association with the ability to empathize with others [47], but it has often been considered as a risk factor in child and adolescent development [48,49]. Like empathic concern, perception of social rejection might interact with stressful life events such as coercive parenting to facilitate a conditional effect of these events on adolescent developmental outcomes, including adolescent delinquent behavior, psychological well-being and educational performances. Indeed, prior research has found that the impact of perceived social rejection is benign or less negative when parents are supportive but becomes more detrimental to child and adolescent wellbeing under the condition of hash or coercive parenting [50].

In traditional Chinese society, coercive parenting is commonly seen as necessary and instrumental in promoting obedience, social conformity and academic achievement when combined with appropriate monitoring and guidance [15]. Prior research has shown that parents in indigenous Chinese communities are more inclined to endorse the use of coercive strategies in raising their children than their counterparts in the West [51]. Studies also found that Chinese adolescents were more accepting of these practices and might interpret parents’ coercive control as a caring act and a source of support [10,11]. Considering the cultural differences, coercive parenting may have no or less negative effect on Chinese adolescents’ academic and social functioning when it is practiced at a reasonable level, especially with children who have a higher need to bond with their parents due to the perception of social rejection outside the home. When adolescents feel socially rejected, a reasonable level of parental attention, even if it is imparted through coercive tactics, can compensate for the lack of psychological and social support outside the home. Moreover, adolescents who have experienced social rejection might be more willing to reveal themselves to their parents, leading to rewarding parent–child interactions, which are particularly beneficial to the development of prosocial behavior and school commitment [52]. However, when coercive parenting reaches a very high level, it might be increasingly difficult for adolescents, including those with strong perception of social rejection, to bond with parents due to repeated exposure to physical and psychological abuse, thereby leading to an elevated risk of delinquency and a reduced level of school commitment [4].

## 2. The Current Study

Prior research has rarely examined the moderated effects of coercive parenting on child and adolescent developmental outcomes. The literature reviewed above offers some indication that the influences of coercive parenting may not stay constant across different adolescent groups. There are strong reasons to believe that adolescents’ empathic concern and perception of social rejection moderate the relationship between coercive parenting and psychosocial outcomes. This position is consistent with the propositions of the differential susceptibility hypothesis and is supported by mounting evidence that the impact of external stressors such as harsh parenting on adolescent development varies with children’s’ affective and cognitive attributes. Hence, contrary to the commonly held belief that coercive parenting has the same effects on children and adolescents across Chinese communities, this study proposes a more complex model postulating that the relationship between coercive parenting and developmental outcomes differs among adolescents with different affective and cognitive attributes within the same communities.

Based on the theories and evidence reviewed earlier, we propose the following hypotheses. First, we expect that coercive parenting remains highly prevalent in Chinese society because it has been an enduring cultural tradition and it is still widely accepted through much of the society, as demonstrated in many previous studies. Second, coercive parenting positively predicts negative developmental outcomes among Chinese adolescents, including delinquency, depression and poor school commitment. Third, we expect empathic concern to moderate the relationship between coercive parenting and adolescent developmental outcomes. Specifically, adolescents with a higher tendency for empathic concern have more positive outcomes when the level of coercive parenting is low or moderate but have poorer outcomes when experiencing a high level of coercive parenting. Fourth, similar to how empathic concern moderates the relationship between coercive parenting and adolescent development, we expect adolescents who have a stronger perception of social rejection to attain better outcomes under low to medium levels of coercive parenting, but develop worse outcomes when exposed to a high level of coercive parenting. Using the two-wave survey data collected from a probability sample of 1085 secondary school students in China, we conducted a series of regression analyses and moderation analyses to test these hypotheses.

## 3. Materials and Methods

### 3.1. Data

The current study used data collected from a two-wave longitudinal study on family processes and delinquency conducted in one of the largest metropolitan areas in China. This study underwent human subject review and was approved by the Research Ethics Committee of University of Macau on 19th December in 2014 (Project identification code is MYRG2014-00120-FSS). We collected the first wave of data in 2015 and the second wave of data one year later. The research site had been a major city in China before the country opened up its economy to the world in the late 1970s, but it has developed into a highly populated and diverse metropolis in recent years with mixed urban and suburban districts. It is now home to 30 million people, including millions of migrant workers and ethnic minorities.

To ensure the representativeness of the sample, we randomly selected eligible participants for the study using a three-stage stratified probability proportionate-to-size sampling procedure. In the first stage, we randomly selected three districts to study, including two urban districts, and one suburban district. In the second stage, we randomly selected one suburban middle school, one urban middle school, one suburban high school, and one urban high school within each district, resulting in a total of 12 schools. In the third stage, in each sampled school, we proportionately selected a random number of classes in the seventh, eighth, tenth, and eleventh grades. Considering that ninth and twelfth graders, which were the final years of middle and high school respectively, would graduate before the second wave of the survey, we did not include them in the baseline survey.

Prior to survey administration, we provided the schools with the written informed consent forms for both the students and their parents. The forms clearly state that the participation in this study is entirely voluntary, and the privacy and confidentiality of the respondents will be strictly protected. Only students who agreed to participate in both waves of the study and whose parents signed a consent form were included in the current study, which yielded 1300 eligible participants. A paper-and-pencil survey was then administered to the sampled students. In the following year (2016), we conducted the second wave of the survey to the same students in the same schools. The response rates for the Wave 1 and Wave 2 surveys were 97.20% and 96.73%, respectively. Additionally, 215 participants who had missing values on study variables, including the non-respondents, were excluded in the analyses, resulting in a final sample of 1085.

The age of the final sample ranged between 11 and 16 at wave 1, with mean age being about 14 years. Approximately 22.5 percent of the respondents were younger than 13 and 21.6 percent were older than 15. The sample was about equally split by gender.

### 3.2. Measurement

The key variables included in this analysis were coercive parenting, empathic concern, perceived social rejection, depression, school commitment, and delinquency. To the greatest extent possible, we used standard instruments with demonstrated reliability and validity to measure these concepts. To facilitate time order, we used data collected in the baseline survey to measure coercive parenting and measures collected in the follow-up survey to measure developmental outcomes, including school commitment, delinquency and depression. We included age, gender and urban residence as control variables in the regression analyses. We also included parental education and family income as control variables in earlier models but dropped them after finding out that their regression coefficients were not statistically significant across the models.

*Coercive parenting*. Coercive parenting was measured by a total of fourteen items, including six questions (the first six listed below) adopted from the National Longitudinal Survey of Youth 1997 [53] and eight questions specifically formulated to measure parental coercive behaviors in the research setting. Each adolescent was asked to rate his or her father/father figure and mother/mother figure separately on a 5-point scale ranging from 1 (never) to 5 (always). The items included “Dad/mom beats you for no reason”, “Dad/mom beats you for your wrongdoing”, “Dad/mom admonishes you in front of other people”, “Dad/mom forbids you to do what you want to do if you do something wrong”, “Dad/mom nags you or scolds you if you make a mistake”, “Dad/mom tells you that you are breaking his/her heart if you do something wrong”, and “Dad/mom nicknames you if you do something wrong”. The Cronbach’s α value of the fourteen items was 0.82, indicating a high level of reliability. We combined scores of both father/father figure and mother/mother figure and used the mean score of the fourteen items as the measure of coercive parenting.

*Empathic concern.* Empathic concern was measured by the empathic concern subscale of the Interpersonal Reactivity Index developed by Davis [35], which has shown strong reliability and validity in Chinese populations [7]. The respondent was asked to rate how well each of the seven statements describes them on a 5-point scale ranging from 0 (does not describe me well) to 4 (describes me very well). Sample items include: “I often have tender, concerned feelings for people less fortunate than me”, “When I see someone being taken advantage of, I feel kind of protective towards them”, and “I am often quite touched by things that I see happen”. We used the centralized sum of the seven items as the measure of empathic concern [54].

*Perceived social rejection.* Perceived social rejection was measured by a reverse-coded version of the social acceptance subscale of the Student Self-Concept Inventory developed by Fleming and Courtney [46]. The Student Self-Concept Inventory has been widely used to measure adolescents’ self-concept and has been shown to have good reliability and validity [55]. In our study, Cronbach’s α value of the subscale was 0.78. Sample items include “You worry about whether other people will regard you as a success or a failure in your job or in school”, “When you think that some people you meet might have an unfavorable opinion of you, you feel concerned or worried about it”, and “You feel concerned about what other people think of you”. The response categories for each item ranged from 1 (practically never) to 7 (very often). As suggested by Fleming and Whalen [56], we used the sum of the five items as the measure of perceived social rejection.

*Depression.* Depression was measured by Center for Epidemiologic Studies Depression Scale (CES-D) developed by Radloff [57]. CES-D consists of 20 items and has shown to have high reliability and validity [58]. In our study, Cronbach’s α value of the 20 items was 0.90, indicating a high level of reliability. Each respondent was asked to rate how many days he/she has the stated depressive symptoms during the past week on a 4-point scale ranging from 1 (less than 1 day) to 4 (5–7 days). Sample items include “I was bothered by things that usually don’t bother me”, “I did not feel like eating; my appetite was poor”, “I felt that I could not shake off the blues even with help from my family or friends”, and “I had trouble keeping my mind on what I was doing”. We used the mean score of the 20 items as the measure of depression.

*School commitment.* School commitment was measured by five items selected from the social bond scale developed by Hirschi [59]. Items include: “You like school”, “It is important to get good grades to you personally”, “You finish your homework”, “You care what teachers think of you” and “The things I do in school seem interesting to me”. The respondent rated each item on a 5-point scale ranging from 1 (never) to 5 (always). The Cronbach’s α value of the 5 items was 0.69. We used the mean score of the five items as the measure of school commitment.

*Delinquency.* Delinquent behavior was measured by the sum of sixteen dichotomous items asking respondents whether they had been involved in delinquent acts within a year. The 18 items included using drugs, selling drugs, fighting with others, threatening someone with weapons, hurting someone with weapons, running away from home, stealing something worth less than 500 RMB (USD70), snatching property from others, committing vandalism, bringing a knife to school, beating or threatening to beat someone, seriously injuring someone, stealing something worth more than 500 RMB, robbery, and joining gang. The response categories for each item were ‘1′ for ‘Yes’ and ‘0′ for ‘No’. The range of delinquency behavior was from 0 to 16.

*Control variables.* Control variables included age, gender and urban residence. Gender (1 = female, 0 = male) and urban residence (1 = urban, 0 = nonurban) were dichotomous variables. Age was an interval variable measured by year, ranging from 9 to 16.

### 3.3. Analytical Approach

To test the research hypotheses, we performed a descriptive analysis to provide an overview of the distributions of the key variables. Second, Pearson’s correlation test was conducted to evaluate the bivariate correlations of the variables. Third, regression analysis was applied to test whether coercive parenting is significantly related to juvenile delinquency, depression, and school commitment. The interaction terms of empathic concern and perceived social rejection with coercive parenting were added in the regression model to examine the possible moderating effects of both variables. To facilitate the interpretation of the results of the moderating effects, the effects of coercive parenting on the three developmental outcomes were plotted under different levels of empathic concern and perceived social rejection. It should be noted that, for different outcomes, we applied different modeling strategies. We applied negative binomial regression to estimate the effects of explanatory variables on juvenile delinquency, as delinquency was measured by an over-dispersed discrete variable. OLS regression was employed to estimate the effects of explanatory variables on depression and school commitment, since both variables were measured by continuous variables. A p value less than 0.05 was taken as the threshold for statistical significance in the current study. STATA 15.1 was used to perform all the statistical analyses. Considering that the percentage of missing values is small (less than 20%), we applied case-wise deletion to deal with unit and item non-responses.

## 4. Results

Table 1 provides the descriptive statistics and bivariate correlations of the variables included in this study. As shown in the table, female respondents accounted for 49.95% of the total sample and the average age was approximately 14 years old. Judging by the averages scores, the respondents reported moderate levels of coercive parenting and moderately high levels of empathic concern and perceived social rejection. As a whole, the adolescents in the sample reported relatively low levels of delinquency and depression, and a moderate level of school commitment.

Table 2 provides some additional information on the distribution of coercive parenting. To show the prevalence of the parenting style, 14 items on coercive parenting were dummy coded and summed up. The sums reflect the types of coercive parenting experienced by the respondents. As shown in the table, very few (1.11%) respondents experienced no incidence of coercive parenting. The mode (24.52%) was located at 7–8 types. Over 85% of the adolescents experienced 5 or more types of coercive parenting. Furthermore, 4.70% experienced more than 12 types of coercive parenting. These results confirm the expectation of relatively high prevalence of coercive parenting in Chinese society.

Table 3, Table 4 and Table 5 present the results of the regression analysis for each of the outcome variables. Three models were estimated for each outcome variable. The first model estimated the main effect of coercive parenting, moderators and other controlling variables on the outcome variable. The second and the third model added the interaction term of coercive parenting and empathic concern and the interaction term of coercive parenting and perceived social rejection respectively to investigate the possible moderating effect of the two moderators.

As observed from the first model in Table 3, both coercive parenting (b = 0.44, *p* < 0.001) and empathic concern (b = −0.06, *p* < 0.001) significantly predicted delinquency. In model two, the effect of the interaction term between coercive parenting and empathic concern was positive (b = 0.06, *p* < 0.05), suggesting empathic concern might elevate the effect of coercive parenting on delinquency. Similarly, in model three, the coefficient of the interaction term between coercive parenting and perceived social rejection (b = 0.03, *p* < 0.05) was also positive, indicating that perceived social rejection might strengthen the effect of coercive parenting on delinquency.

Table 4 lists the regression coefficients of the explanatory variables on school commitment. As shown, the main effect of coercive parenting was not statistically significant in any of the models. However, empathic concern (b = 0.04, *p* < 0.001) and perceived social rejection (b = 0.02, *p* < 0.001) were significantly related to school commitment. The two interaction terms, moreover, were both significant as shown in Model 2 and Model 3. The negative effects of the interaction terms between coercive parenting and empathic concern (b = −0.02, *p* < 0.01) as well as the interaction terms between coercive parenting and perceived social rejection (b = −0.01, *p* < 0.05) indicated that empathic concern and perceived social rejection might weaken the negative effect of coercive parenting on school commitment.

Table 5 presents the regression coefficients of the explanatory variables on depression. It shows that the main effects of coercive parenting (b = 0.13, *p* < 0.001) and perceived social rejection (b = 0.02, *p* < 0.001) were both positive and statistically significant, suggesting that these two variables might increase depression. However, the main effect of empathic concern was negative, indicating that it might reduce depression (b = −0.01, *p* < 0.001). With regard to the moderating effect, only the interaction terms of coercive parenting and perceived social rejection were significant (b = 0.01, *p* < 0.01), suggesting that coercive parenting might have stronger negative effect on depression with higher levels of perceived social rejection.

**Figure 1:**
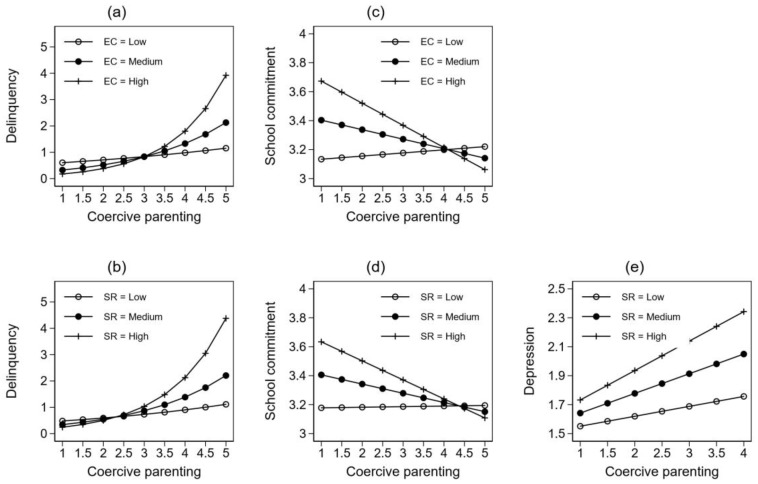
The marginal effect of coercive parenting on developmental outcomes under different levels of empathic concern (EC) and perceived social rejection (SR). (**a**) Moderated relationship between CP and delinquency by EC. (**b**) Moderated relationship between CP and delinquency by SR. (**c**) Moderated relationship between CP and school commitment by EC. (**d**) Moderated relationship between CP and school commitment by SR. (**e**) Moderated relationship between CP and depression by SR.

The results of regression analysis indicate that the two moderators, empathic concern and perceived social rejection, significantly moderated the relationships between coercive parenting and the three outcome variables. To facilitate the interpretation of the moderating effects, we simulated the values of coercive parenting from its lowest to the highest value and predicted the corresponding value of the outcome variables on three levels of the moderators: low (average value − 1 standard deviation of the moderator), medium (average value) and high (average value + 1 standard deviation). Figure 1 illustrates the patterns of relationships between coercive parenting and the outcome measures at different levels of empathetic concern (EC) and perceived social rejection (SR). It should be noted that since negative binomial regression was applied to model the effect of the explanatory variables on delinquency, dependent variables were logarithm transformed. Consequently, the relationship between coercive parenting and delinquency was not linear.

Figure 1a shows the effect of coercive parenting on delinquent behavior under different levels of EC. Overall, coercive parenting was positively related to delinquency, but the slope varied across different levels of EC: Individuals with higher EC (i.e., high and average EC) were shown to be more sensitive to the influence of coercive parenting as demonstrated by the slopes. Moreover, compared to the low EC group, those with higher EC had lower delinquency before the crossover point between 3 and 3.5 on coercive parenting. After the crossover point, the positive effect of coercive parenting on delinquency increased rapidly. These results suggest that EC might mitigate the positive effect of coercive parenting on delinquency as long as coercive parenting was not too high.

Figure 1b shows similar patterns. As in Figure 1a, coercive parenting was positively related to delinquency. Moreover, the slope of coercive parenting also varied across different levels of SR and the rates of change differed similarly before and after crossover point. The main difference between Figure 1b and Figure 1a is the placement of the crossover point. This crossover point for SR is between 2 and 2.5 on coercive parenting, suggesting that the turning point for the effect of coercive parenting arrived earlier in reference to the value of SR, compared to the value of EC. Specifically, the adolescents with higher SR began to show higher involvement in delinquency than those with low SR when coercive parenting reached above the range of 2 and 2.5.

Figure 1c illustrates the patterns of the effect of coercive parenting on school commitment at different levels of EC. Overall, coercive parenting decreased school commitment at higher levels of EC but increased school commitment at lower levels of EC. However, before the crossover point at around 4 on coercive parenting, the two groups with higher EC actually had higher school commitment. Judged by this, EC mitigated the negative effect of coercive parenting on school commitment until it reached a very high level. The rates of change for the two groups of adolescents with higher levels of EC were greater as demonstrated by the slope of the straight lines, indicating that the groups with higher EC were more susceptible to the influence of coercive parenting.

As in Figure 1c, similar patterns of interaction were found between coercive parenting and SR with regard to school commitment (see Figure 1d). Overall, the slopes showed that coercive parenting decreased school commitment for the groups with high and medium SR but increased school commitment among the group with low SR. Before the crossover point at about 4.5 on coercive parenting, the two groups with higher SR had higher school commitment, suggesting that SR alleviated the negative impact of coercive parenting on school commitment under the conditions of low and moderate coercive parenting. However, the two groups with medium and high SR were more susceptible to the influence of coercive parenting, as shown by the rates of change in the slopes associated with them. As such, school commitment deteriorated more rapidly as perceived coercive parenting became more intense. After the crossover point, the group with higher SR had lower school commitment compared to the groups with lower SR.

Lastly, Figure 1e illustrates the relationship between coercive parenting and depression at different levels of SR. It shows that the relationship between coercive parenting and depression remained positive regardless of the level of perceived social rejection. However, compared with the adolescents with low SR, the groups with medium and high SR showed not only higher starting points in depression but also steeper slopes, indicating that SR might aggravate the positive relationship between coercive parenting and depression.

## 5. Discussion

Coercive parenting is believed to be a prevalent parenting style in Chinese society. Proponents of this parenting style stress its positive role in fostering social conformity and academic achievement. Critics, on the other hand, underline its detrimental impact on autonomy development and psychological wellbeing. It is our contention that both of these arguments may be overly simplistic. Based on the differential susceptibility hypothesis, we argue that the impact of coercive parenting on adolescent developmental outcomes may vary depending on the characteristics of the adolescent. In this study, we first assessed the prevalence of coercive parenting in a probability sample of adolescents drawn from one of the largest urban areas in China. We then used the two-wave data to test the moderating effects of adolescent empathic concern and perception of social rejection on the relationship between coercive parenting and developmental outcomes across three major psychosocial domains of adolescent development, including delinquency, academic commitment, and mental health.

The adolescents in this study reported widespread coercive behavior from their parents. More than 85% of them experienced five or more types of coercive parenting behavior. The average score of coercive parenting is 2.13 on a five-point scale, indicating an experience that was more than occasional or more than limited. These results confirm the first hypothesis that coercive parenting remains highly prevalent in Chinese society. This study also finds partial support for the second hypothesis by demonstrating that coercive parenting positively predicted negative developmental outcomes including delinquency and depression among Chinese adolescents. In addition, the findings lend strong support for the third and fourth hypotheses of the moderating effects of adolescent empathic concern and perception of social rejection with regard to delinquency and school commitment. Consistent with the differential susceptibility hypothesis, both adolescent empathic concern and perception of social rejection appeared to increase the adolescent’s plasticity to the influence of coercive parenting. Compared to those who were low on empathic concern and perceived social rejection, adolescents with more of these attributes were more susceptible to the influence of coercive parenting at both low and high ends of parenting behavior. Specifically, adolescents with higher empathic concern and perceived social rejection exhibited lower delinquency and higher academic commitment than those who were low on these two attributes under the conditions of low and medium coercive parenting. However, when coercive parenting reached the high-to-extreme level, the adolescents with strong empathic concern and perceived social rejection exhibited worse developmental outcomes in delinquency and school commitment.

There are several plausible explanations for the moderating effects of empathic concern and perceived social rejection on the relationship between coercive parenting and developmental outcomes in Chinese society. First, adolescent empathic concern and perception of social rejection are related psychological characteristics as they both reflect shyness and social anxiety [33,60]. For this reason, it is not surprising that they demonstrated similar moderating effects in the context of coercive parenting. Second, empathic concern represents the feelings of compassion and concern for others in need or distress. Adolescents who had a stronger capacity for empathic concern might be more sympathetic to parental coercive behavior although this type of behavior could be stressful and objectionable. These adolescents had the ability to establish an emotional connection with their parents and developed a positive child-parent relationship, which could operate as a protective factor against delinquency and poor academic outcomes under the condition of moderate to normal coercive control. They were, however, more sensitive to how others treated them because of their elevated susceptibility to external influences. When coercive parenting reached an unbearable level, it would affect these adolescents more negatively than those with lower levels of empathic concern. Third, adolescents with higher perceived social rejection also tended to be more susceptible to the impact of external stressors such as social exclusion and abusive parenting. At the same time, these adolescents were also in higher need of emotional and social support. While coercive parenting might not be ideal, it might not be overly negative either when practiced in moderation in Chinese society where adolescents have a higher tendency to interpret parental coercive behavior as acts of love and caring [9,11]. In this context, adolescents might be able to find comfort and support in their parents who attended to their needs despite being coercive to a certain degree, especially when they perceived strong rejection in social relationships outside the home. These patterns of interaction between parents and the adolescent under the stress of social rejection might lead to positive developmental outcomes in delinquency prevention and school commitment. On the other hand, when coercive parenting became excessive, its negative impact would be so strong that it would cease to be a source of support for adolescents experiencing social rejection. Under these circumstances, adolescents with a strong perception of social rejection would be especially vulnerable because they would be negatively impacted by both social rejection and excessive coercive parenting behavior.

With regard to depression, the patterns of relationship were quite different from those for the other two developmental outcomes. The positive relationship between coercive parenting and depression did not vary by empathic concern, suggesting that coercive parenting increased depression at a constant rate regardless of the level of empathic concern. On the other hand, the relationship showed some fluctuation depending on the magnitude of perceived social rejection. Overall, coercive parenting was positively related to depression at all levels of perceived social rejection. However, the strength of the relationship varied at different levels of perceived social rejection. The higher the perception of social rejection, the more strongly coercive parenting predicted depression. Therefore, coercive parenting appeared to have a more detrimental effect on mental health among adolescents who had elevated levels of perceived social rejection.

The findings of this study have important research and practice implications. In the last several decades, researchers have devoted considerable efforts to identifying a specific parenting style that can explain the observed patterns of psychosocial adjustment of Chinese children [1,15]. There has been general agreement that Chinese parenting as shaped by Confucian teaching is more control-oriented and more coercive than typical Western parenting. Further, as argued by the sociocultural perspective commonly adopted in the research [51,61], this style of parenting is supposed to have similar effects in all Chinese families, particularly with respect to the promotion of culturally desirable goals of social conformity and academic achievement. Whereas this study lends support for the view that coercive parenting is prevalent in China, it offers strong evidence contradicting the proposition that coercive parenting may influence Chinese adolescents in similar ways. The findings of the moderating effects of adolescent empathic concern and perception of social rejection call attention to individual characteristics that may condition the impact of coercive parenting. As such, the current study should contribute to a more in-depth and contextualized understanding of how coercive parenting shapes adolescent development. It also establishes the importance of examining the dynamic interactions among multiple systems when assessing the influences of parenting practices on adolescent development in empirical research.

In terms of practice implications, the current study demonstrates the need to consider individual differences when providing services to adolescents impacted by negative or ineffective parenting practices. The research findings show that the influence of coercive parenting on delinquency or school commitment was a function not only of the level of parental coercive control but also its interactions with empathic concern and perceived social rejection. Therefore, parenting programs intended to reduce delinquency and to strengthen academic commitment should consider these dynamic processes and develop individualized programs that effectively address the specific needs of different groups of adolescents. It might be too simplistic to assume that all adolescents from a specific social and cultural background, for example, at-risk adolescents in Chinese society, respond to certain parenting style in the same way. Similarly, programs designed to prevent adolescent mental health problems should consider how coercive parenting might interact with individual characteristics to foster mental illness such as depression. The current study indicates that no level of coercive parenting might be safe for adolescent mental health development. Moreover, coercive parenting might be especially harmful for adolescents who experienced social rejection by elevating their risks for depressive symptoms over and above the stress they have already experienced in social relationships outside the home. Hence, mental health programs for adolescents should address the dual risk factors of coercive parenting and social rejection.

Despite the contributions that it has made to the understanding of the conditional effects of coercive parenting in Chinese society, the current study has several major limitations that should be noted. First, the sample generated for this study consisted of students from secondary schools in one city in China. It is unclear if the patterns observed in this study is generalizable to other cities in China. Second, due to lack of data, this study did not control for biological factors such as genetic dispositions for psychological and behavioral maladjustment. As these factors may influence adolescents’ responses to parenting practices and developmental outcomes, the omission of these variables might have had a negative impact on the accuracy of our findings. Third, the measure of coercive parenting used in this study was based on the children’s perceptions using self-report data. Although children’s perceptions represent a reasonable way to measure parenting styles and their impact on children [62], they may be biased by children’s own interpretation of the behavior or event in question. To address these limitations, future research should use probability samples drawn from multiple geographical areas to validate the research findings. Furthermore, to the extent possible, they should include measures of genetic factors related to key explanatory and/or outcome variables to control for the confounding effects. Additionally, future studies should consider incorporating reports of multi-informants such as parents, teachers and peers to improve the reliability of the measurement of parenting practices.

## 6. Conclusions

Through the analysis of the two-wave data collected from a probability sample of 1085 adolescents, the present study showed that coercive parenting remained highly prevalent in Chinese society. We found that the relationship between coercive parenting and adolescent developmental outcomes varied depending on not only the adolescents’ levels of empathic concern and perception of social rejection but also the specific domain of the outcome examined. Adolescents with higher levels of empathic concern and perceived social rejection exhibited less delinquency and stronger academic commitment than those with lower levels of empathic concern and perceived social rejection, under the condition of low-to-moderate coercive parenting. However, as coercive parenting increased to a high level, the adolescents with stronger empathic concern and perceived social rejection started showing worse developmental outcomes with regard to delinquency and school commitment. In contrast, coercive parenting showed a more consistent positive relationship with depression, which was slightly moderated by the perception of social rejection but was invariant across different levels of adolescent empathic concern.

## Figures and Tables

**Table 1:** Descriptive statistics and bivariate correlations of study variables (*N* = 1085).

Variables	(4)	(5)	(6)	(7)	(8)	(9)	M/%	SD
1. Age							13.83	1.49
2. Female							49.95%	
3. Urban residence							72.26%	0.45
4. Coercive parenting	1.00						2.13	0.63
5. Empathic concern	−0.02	1.00					4.33	4.75
6. Social rejection	0.08 **	0.18 ***	1.00				19.83	7.49
7. Delinquency	0.09 **	−0.11 ***	−0.03	1.00			0.61	1.69
8. School commitment	−0.03	−0.27 ***	0.23 ***	−0.18 ***	1.00		3.33	0.75
9. Depression	0.16 ***	−0.06 *	0.28 ***	0.14 ***	−0.18 ***	1.00	1.80	0.60

Note: * *p* < 0.05, ** *p* < 0.01, *** *p* < 0.001.

**Table 2:** Type of coercive parenting experienced by the adolescents.

Coercive Parenting	*N*	%	Cumulative%
0	12	1.11	1.11
1–2	42	3.87	4.98
3–4	106	9.77	14.75
5–6	212	19.53	34.29
7–8	265	24.42	58.71
9–10	242	22.30	81.01
11–12	155	14.29	95.30
13–14	51	4.70	100.00

**Table 3:** Regression coefficients of explanatory variables on delinquency.

Variables	(1)	(2)	(3)
	DV: Delinquency	DV: Delinquency	DV: Delinquency
Age	−0.03	−0.02	−0.03
	(0.05)	(0.05)	(0.05)
Female	−0.96 ***	−0.97 ***	−1.01 ***
	(0.15)	(0.15)	(0.15)
Urban residence	−0.13	−0.13	−0.15
	(0.16)	(0.16)	(0.16)
Coercive parenting (CP)	0.44 ***	0.19	−0.21
	(0.12)	(0.15)	(0.30)
Empathic concern (EC)	−0.06 ***	−0.20 ***	−0.05 ***
	(0.02)	(0.06)	(0.02)
Social rejection (SR)	−0.00	−0.00	−0.08 *
	(0.01)	(0.01)	(0.03)
CP×EC		0.06 *	
		(0.03)	
CP×SR			0.03 *
			(0.01)
Constant	−0.33	0.00	1.07
	(0.77)	(0.78)	(0.98)
Alpha	1.19 ***	1.17 ***	1.17 ***
	(0.10)	(0.10)	(0.10)
N	1085	1085	1085

Note: *p*-values in parentheses; * *p* < 0.05, ** *p* < 0.01, *** *p* < 0.001.

**Table 4:** Regression coefficients of explanatory variables on school commitment.

Variables	(1)	(2)	(3)
	DV: School Commitment	DV: School Commitment	DV: School Commitment
Age	−0.05 ***	−0.05 ***	−0.05 ***
	(0.01)	(0.01)	(0.01)
Female	0.03	0.03	0.04
	(0.04)	(0.04)	(0.04)
Urban residence	0.02	0.02	0.02
	(0.05)	(0.05)	(0.05)
Coercive parenting (CP)	−0.06	0.01	0.12
	(0.03)	(0.04)	(0.09)
Empathic concern (EC)	0.04 ***	0.08 ***	0.04 ***
	(0.00)	(0.02)	(0.00)
Social rejection (SR)	0.02 ***	0.02 ***	0.04 ***
	(0.00)	(0.00)	(0.01)
CP×EC		−0.02 **	
		(0.01)	
CP×SR			−0.01 *
			(0.00)
Constant	3.61 ***	3.46 ***	3.22 ***
	(0.23)	(0.24)	(0.29)
N	1085	1085	1085
R^2^	0.12	0.13	0.12

Note: *p*-values in parentheses; * *p* < 0.05, ** *p* < 0.01, *** *p* < 0.001.

**Table 5:** Regression coefficients of explanatory variables on depression.

Variables	(1)	(2)	(3)
	DV: Depression	DV: Depression	DV: Depression
Age	0.01	0.01	0.01
	(0.01)	(0.01)	(0.01)
Female	0.02	0.02	0.02
	(0.04)	(0.04)	(0.04)
Urban residence	0.02	0.02	0.01
	(0.04)	(0.04)	(0.04)
Coercive parenting (CP)	0.13 ***	0.11 **	-0.04
	(0.03)	(0.04)	(0.07)
Empathic concern (EC)	−0.01 ***	−0.02 *	−0.01 ***
	(0.00)	(0.01)	(0.00)
Social rejection (SR)	0.02 ***	0.02 ***	0.00
	(0.00)	(0.00)	(0.01)
CP×EC		0.00	
		(0.01)	
CP×SR			0.01 **
			(0.00)
Constant	0.94 ***	0.98 ***	1.33 ***
	(0.18)	(0.19)	(0.23)
N	1085	1085	1085
R^2^	0.11	0.11	0.12

Note: *p*-values in parentheses; * *p* < 0.05, ** *p* < 0.01, *** *p* < 0.001.
